# Forward Osmosis as Concentration Process: Review of Opportunities and Challenges

**DOI:** 10.3390/membranes10100284

**Published:** 2020-10-14

**Authors:** Gaetan Blandin, Federico Ferrari, Geoffroy Lesage, Pierre Le-Clech, Marc Héran, Xavier Martinez-Lladó

**Affiliations:** 1 Eurecat, Centre Tecnològic de Catalunya, Water, Air and Soil Unit, 08242 Manresa, Spain; xavier.martinez@eurecat.org; 2 Institut Européen des Membranes, IEM, Université de Montpellier, CNRS, ENSCM, 34090 Montpellier, France; geoffroy.lesage@umontpellier.fr (G.L.); marc.heran@umontpellier.fr (M.H.); 3 Catalan Institute for Water Research (ICRA), 17003 Girona, Spain; fferrari@icra.cat; 4 UNESCO Centre for Membrane Science and Technology, School of Chemical Engineering, University of New South Wales (UNSW), Sydney, NSW 2052, Australia; p.le-clech@unsw.edu.au

**Keywords:** cold concentration, food concentration, nutrients recovery, water reuse, brine concentration, osmotic process

## Abstract

In the past few years, osmotic membrane systems, such as forward osmosis (FO), have gained popularity as “soft” concentration processes. FO has unique properties by combining high rejection rate and low fouling propensity and can be operated without significant pressure or temperature gradient, and therefore can be considered as a potential candidate for a broad range of concentration applications where current technologies still suffer from critical limitations. This review extensively compiles and critically assesses recent considerations of FO as a concentration process for applications, including food and beverages, organics value added compounds, water reuse and nutrients recovery, treatment of waste streams and brine management. Specific requirements for the concentration process regarding the evaluation of concentration factor, modules and design and process operation, draw selection and fouling aspects are also described. Encouraging potential is demonstrated to concentrate streams more than 20-fold with high rejection rate of most compounds and preservation of added value products. For applications dealing with highly concentrated or complex streams, FO still features lower propensity to fouling compared to other membranes technologies along with good versatility and robustness. However, further assessments on lab and pilot scales are expected to better define the achievable concentration factor, rejection and effective concentration of valuable compounds and to clearly demonstrate process limitations (such as fouling or clogging) when reaching high concentration rate. Another important consideration is the draw solution selection and its recovery that should be in line with application needs (i.e., food compatible draw for food and beverage applications, high osmotic pressure for brine management, etc.) and be economically competitive.

## 1. Introduction

### 1.1. State of the Art Concentration Processes

Concentration processes are part of the main operation units in many industrial sectors, such as food processing, mineral extraction, chemicals processing or environmental applications. Various processes exist and their selection depends both on the properties of the stream to be concentrated, requirements of the applications and final products. Those processes are classified in two main categories—membrane and thermal concentration processes. Among membrane processes, microfiltration (MF) and ultrafiltration (UF) are very broadly used and considered state of the art separation technologies in various applications (food industry, chemical industries, water treatment, etc.) [1]. These technologies are of interest for all concentration processes that require the concentration of molecules and particles with particle sizes above 0.01 and up to 1 µm. Typically, pathogens (bacteria, viruses, etc.), organic macromolecules (proteins, carbohydrates, etc.), and minerals (clays, latex, etc.) can be well rejected (and extracted or concentrated) by microporous membranes. Its main advantage is the relatively low operating pressure (from 0.1 to 5 bar) and therefore limited energy costs. High pressure membrane processes, such as reverse osmosis (RO) retain the smallest organics (i.e., tannins, organic acids, polyphenols, pesticides, pharmaceuticals, etc.), heavy metals and salts. In comparison with thermal processes, RO proves to be less energy intensive, requires less investment costs, has a lower footprint and avoids the thermal degradation of compounds (cooking taste) in food industry [2]. One main disadvantage of RO is its inability to reach high concentration levels due to the hydraulic pressure limitation. Current practices limit RO operational pressure to 80 bar due to mechanical resistance and required energy. Another limitation of RO is the fouling propensity when treating complex stream and given the high pressure operation [3]. As an alternative to UF/MF and RO, nanofiltration (NF) features intermediate properties (operating pressure, molecular weight cut-off) that allow for the rejection of compounds down to divalent ions and with a variety of membrane characteristics that can make it a more adequate choice [4]. Membrane distillation (MD) presents many attractive features, such as lower operating temperatures (<70 °C) compared to thermal processes, compact process, high rejection, not limited by osmotic pressure and possibility to use waste heat [5]. However, MD, so far, remains at the pilot scale, as it is not energetically and economically outperforming existing technologies (even when waste heat is used) and there are no commercially available modules for large scale applications [6]. Osmotic distillation (or osmotic evaporation) is particularly suited for the processing of heat-sensitive aqueous solutions, such as fruit juices and pharmaceutical products. The solution to be concentrated is separated by a porous hydrophobic membrane from a high osmotic pressure solution. OD was mostly tested for food concentration [2,7] but, as for MD so far, it remains on pilot scale. Among the thermal processes, evaporation is an established treatment used in a broad range of applications. The main advantages of evaporation are the relatively low technology grade, low maintenance costs and the possibility to remove high content of water. As main drawbacks, temperature sensitive compounds are altered by the high temperature and evaporation requires high amount of energy to produce heat. As alternatives, solar evaporation allows for operation at lower temperature but needs an extensive surface (lagoons). Cryoconcentration is a natural phenomenon which occurs during ice thawing. A more concentrated phase is then separated from the initial solution. By avoiding high temperature treatment, cryoconcentration technology can produce highly nutritive compounds, with biological and organoleptic value [8]. However, the commercial solution still requires remarkable energy consumption and achievable concentration remains below that of evaporation [9]. Thus, to date, none of the existing technologies have offered a fully satisfying solution allowing for high concentration rate at low energy costs and without affecting the properties of the concentrated products.

### 1.2. The Emergence of Forward Osmosis

**Figure 1:**
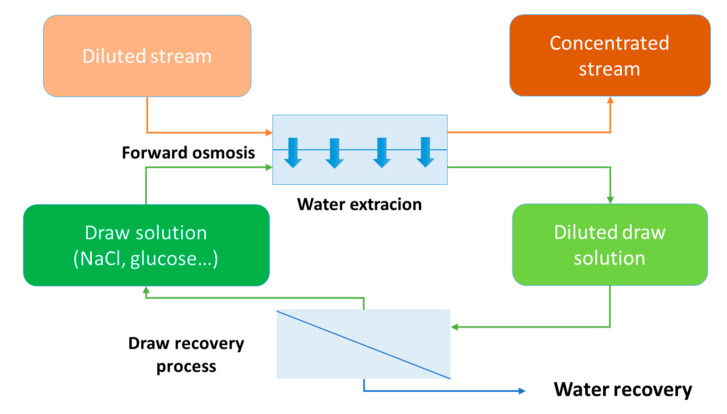
Basic principles of forward osmosis as concentration process.

In forward osmosis (FO), the solute concentration gradient (also called osmotic pressure differential, ∆π) acts as the driving force between two liquids separated by a selectively permeable membrane. As a result, the permeation of water occurs through the membrane from the lowest (feed solution) to the highest solute concentration solutions (so-called draw solution) while suspended solids, most of them dissolved molecules or ions, do not pass through the membrane and are concentrated in the feed (Figure 1). First work of Loeb in the late 1970s [10,11,12] on osmotic processes remained relatively unexplored until new semi-permeable membranes, tailor-made for osmosis applications, were developed in the early 2000s [13,14,15,16]. The two main osmotically driven processes that were initially considered were FO and pressure retarded osmosis (PRO). Both FO and PRO sparked intense research, being seen as novel and highly promising technologies for seawater desalination and energy production, respectively [17,18]. However, following progress in the technology and development from initial laboratory proof of concept towards full scale implementation in those targeted applications, both FO and PRO faced challenges both in terms of technical limitations (low flux, membrane resistance at high pressure for PRO) and clear competitive advantages with regard to initial applications forecasted [19,20]. Thus, and despite significative advances in membrane and modules designs and operation, so far those applications of osmotic processes remain mostly at the pilot scale. Intense research following the FO boom led, however, to broaden the interest and scope of potential FO applications. Several specific reviews discussed the interests, principles, limitations and challenges for future development of the FO process. These reviews focused on mass transfer limitations including internal and external concentration polarization (ECP and ICP) [17,21,22], membrane developments [21,23], fouling [24], rejection of trace organic contaminants [25], optimized draw solutions [21,26,27], energy aspects [19,22] and potential applications [19,28,29,30], including WW treatment [31], desalination [27] and the hybridization of FO with other processes which will not be extensively reviewed in this manuscript [29].

Unique properties of FO rely on the following features [23,27,31,32,33]: (1) dense membrane with high selectivity and rejection of most compounds (like reverse osmosis (RO) membrane) enable us to only (mostly) extract water from the feed leading to high concentration rate; (2) no hydraulic pressure is required for water permeation, making the FO process as a soft technology; (3) the osmotic driven force allows for easier operation and does not require pressure vessels and expensive high pressure pumps, FO can even be operated in submerged mode offering several design possibilities; (4) no temperature gradient is required, which also avoids the degradation of feed compounds and at minimised energy costs (FO is also called cold concentration); (5) possible operation at very high osmotic pressure allowing us to treat already concentrated streams and reaching high concentration ratios; (6) treatment of viscous or charged/challenging feeds due to the lack of pressure or operation in submerged mode; (7) minimised fouling rate when operated at low flux and easy cleaning since the fouling layer is not compacted on the membrane, as in the pressurised process. Thus, FO can be a preferred candidate for concentration of streams and an alternative to the established concentration processes which have some inherent drawbacks (Table 1).

**Figure 2:**
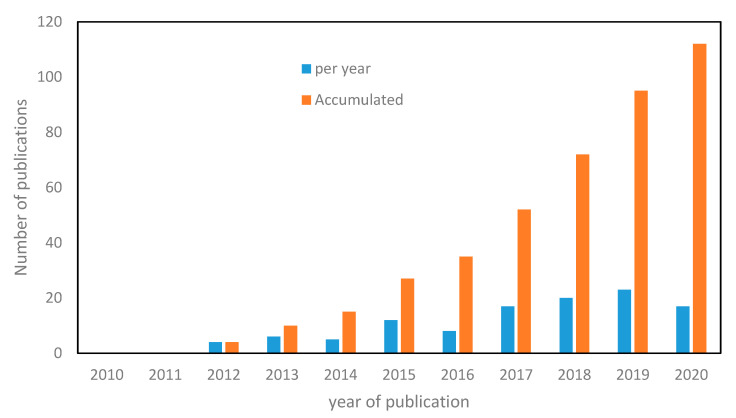
Yearly and accumulated numbers of publication on forward osmosis for concentration (database: Scopus, search parameters: “forward osmosis concentration” in title, abstract and keywords and after removing results from “forward osmosis concentration polarization”.

New applications where current technologies showed limitations for extraction and purification of liquids, such as food concentration or complex streams, such as brines or highly concentrated wastewater (WW) are envisioned for FO [34]. Numerous studies exist regarding concentration with FO for various type of applications (food processing, recovery of value added organics, nutrients recovery from WW streams or concentration of waste streams). After remaining confidential, in the past 10 years growing interests have been shown with regard to the use of forward osmosis as a concentration process (Figure 2) with more than 110 scientific research papers published recently. However, to date, no review has been dedicated to the specific potential of FO as a concentration process. The first part of this review therefore aims at compiling, for the first time, all studies reporting FO operation as concentration process. Then, in the second part, the existing gaps in knowledge both at fundamental and practical levels towards FO implementation as a concentration process, and the possible limitations to be overcome for broader developments of FO as a concentration process, will be critically discussed.

## 2. Applications of FO as Concentrating Process

### 2.1. Food and Beverages

#### 2.1.1. Liquid Food Concentration

Vast amounts of liquid food are industrially concentrated to reduce storage, packaging, handling and transportation costs [35]. Thermal processing remains the most widely employed method for shelf-life extension and food preservation and concentration [36]. However, such technologies suffer from serious drawbacks, such as high energy demand and loss of product quality (colour degradation, “cooking taste”, loss of aroma) and its nutritive properties (vitamins, anthocyanins, carotenoids, etc.) [2]. As an alternative, recent efforts have focused on (non-thermal) membrane processes; with UF and MF being used for clarification or large molecular-weight compounds separation, and NF and especially RO more dedicated to concentration as such, thanks to their high rejection properties. The benefits of RO in comparison with thermal technologies include better preservation of flavours and aroma, lower energy and investment costs. As the main drawback, achieved concentrations remain limited to 25–30° brix due to the high osmotic pressure of the concentrated product and limits in hydraulic pressure operation of RO. Thus, to date, RO remains a pre-concentration process and could not fully compete with evaporation to reach concentration rates of 45–65° brix [2].

The first attempt to use FO for liquid food was in 1966 but remained confidential [37]; only in the 90s with the development of dedicated FO membranes, more intense research was initiated [38]. A broad range of liquid food was concentrated using FO: grape, raspberry, orange, pineapple, kokum, grapefruit, jaboticaba, red radish, tomato, beetroot, coffee, sucrose [36,39,40]. Early studies demonstrated the proof of concept—orange and red raspberry juice were successfully concentrated with high acid and colour rejection (>99.9%). FO concentrates with similar flavour and aroma than commercial products were obtained [16,41]. A significant amount of research on tomato juice concentration by Petrotos and Lazarides [35,42,43] confirmed generally positive results, but FO fluxes were limited to below 4.5 L·m^−2^·h^−1^. Following the development of dedicated FO membrane, several studies demonstrated the possibility to concentrate the following: pineapple juice up to 60° Brix [44], Kokum juice from 2 to 52° Brix [45], beetroot, pineapple and grape juice from 2.3 to 52° Brix, 8.0 to 54.6° Brix and 4.4 to 54° Brix, respectively [46]. Positively, observed permeation fluxes were mostly above 5 L·m^−2^·h^−1^ showing the importance of dedicated FO membrane. Those studies confirmed the beneficial advantage of FO vs. thermal processes in concentrating and avoiding the degradation of valuable components, such as anthocyanin in Kokum, betalain in beetroot juice or ascorbic acid in pineapple. Reconstituted fresh juice from those concentrates also proved to be very similar to initial fresh juice [47]. Sant’Anna et al. confirmed that FO preserved nutraceutical and organoleptic characteristics of the jaboticaba juice unlike thermal concentration, which decreases anthocyanin and compounds with antioxidant capability, beyond of changes in beverage colour properties [40]. Overall beneficial interests in flavour and nutritional properties preservation pushes the FO market towards an even broader range of liquid juice application (coffee concentration, bergamot juice, coconut milk, strawberry juice) [48,49,50].

Among the limitations of FO for food processes, the compatibility of the draw solution with the concentrated food juice needs special attention. Even if the FO membrane rejects most compounds, small fractions of the draw solution may permeate through the membrane and alter product flavours. Mostly sucrose/glucose and NaCl (Table 2) were used as a draw solution, but no study has reported an impact on the juice concentrate. An et al. used potassium sorbate (a common preservative in the food industry) as a draw solution thanks to its high osmotic potential and low reverse permeation flux. In their study, apple juice concentrate flavours were not altered and potassium sorbate in the juice concentrate was in line with legislation [51]. Another limitation is the membrane fouling and enhanced concentration polarisation that may occur especially with complex liquids and in the presence of proteins (pectins, etc.) that can form a gel layer on the membrane surface. The application of ultrasound proved to be beneficial to mitigate gel layer formation in the context of sweetlime juice, and rose extract anthocyanin colorant solution concentration and with minimal degradation of anthocyanin [52]. A recent study used a new generation of highly permeable thin-film composite (TFC) membranes for the concentration of grapefruit juice [39]. Such membrane allowed for significant improvement of permeation flux (>10 L·m^−2^·h^−1^), testifying to recent progress in FO membrane development. Significant fouling propensity was observed but easily mitigated by pre-treatment to remove suspended solids. Additionally, membrane cleaning by simple physical washing allowed for sustainable operation. 

#### 2.1.2. Dairy

Eliminating the water content in milk is an essential step during several dairy product manufacturing processes, such as in cheese, yogurt, concentrated milk and milk powder production [56]. Dairy products are generally concentrated using multi-stage evaporators (concentration, to remove up to 90% of the water) followed by spray-drying if powder is required [57]. For example, concentrated milk dry matter content should be increased from 8–12% to 20–25%; in the milk powder process, milk is concentrated until 50% dry matter content before being sent to the spray dryer [56]. Overall, the production of whey powder and powdered milk require the removal of water from raw milk (85.5–89.5% moisture) to reach 3–5% moisture in the final product [58]. Thermal concentration can affect the functional properties and digestibility of dairy heat-sensitive products and is energy intensive, accounting for the highest share of overall energy needed in the dairy industry [57,59,60]. Membrane technologies were developed for dairy separation/fractionation (UF) and concentration (NF, RO), since their energy demand is much smaller than evaporation [61]. However, given the osmotic pressure of concentrated dairy fractions, and hydraulic pressure to be exerted, membrane concentration can only reach a maximum dry weight of 12–20%. Thus, RO is mostly used as pre-concentration step before evaporation to reach the desired final concentration, especially in the context of whey products [60]. 

So far, FO studies dedicated to dairy processes remain scarce and focused on two steps: milk and whey concentration (Table 3). Only one FO study dedicated to skim milk concentration was reported. In that study performed on an 8´´ pilot scale setup, the concentration objective of 2.5 fold was achieved (from 8 to 21% wt) [62], proving the feasibility to concentrate milk using FO. The passage of small organic molecules was detected, which caused foaming in the draw solution during the concentration process. Moderate fouling was observed in the conditions tested (flux below 10 L·m^−2^·h^−1^) and the cleaning protocol (citric acid followed by enzyme based solution cleaning) was able to completely restore the initial membrane performance.

Current efforts are developed to valorise whey and its valuable compounds through fractionation and concentration steps. Aydiner et al. conducted a series of studies on FO–RO hybrids for whey recovery and concluded that FO–RO can achieve high concentrations of whey (25–35%), high recovery of water for reuse and be economically competitive [63,64,65]. Wang et al., using in-house made TFC HF membranes, also reached 22% dry solids whey thanks to a CF of 3.6 [66]. Chen et al. evaluated whey concentration potential by FO, reaching a CF of 2.5 times and without significant change in dry product composition [62]. This study also pointed out that significant energy savings can be achieved using FO instead of thermal processes or (standalone) RO. Of special interest, the potential use of saline brines (produced during the dairy process) as draw solutions avoids the necessity of draw a recovery step, which bring a significant advantage for FO implementation.

**Table 3:** Review of FO concentration studies for dairy applications.

Application	Membrane Type	Draw Solution	T (°C)	Initial Jw (L·m^−2^·h^−1^)	Initial Conc.	CF	Final Conc.	Ref.
Skim milk	CTA FO	0.9 M NaCl	16–19	4.5	8%	2.8	22%	[62]
Whey	CTA FO	0.9 M NaCl	16–19	4	5.9%	2.5	15%	[62]
Whey	TFC FO	0.5 M NaCl	22.5	12	6%	3.6	22%	[66]
Whey	CTA FO	2 M NaCl	25	28	6.8%	4	28%	[64]
Whey	CTA FO	2 M NaCl	25	12	2.5%	8	21%	[65]
Whey	CTA FO	3 M NaCl	25	20	6.8%	2.1	14%	[63]

#### 2.1.3. Alcohol

Several research groups studied the possibility to separate alcohol, mostly ethanol, from water through FO. The potential interest is both for alcohol purification in the context of its production or the concentration or removal from beverages such as beer or wine. The advantage of FO relies on operation at low temperature, preserving taste and flavours and/or avoiding high energy costs of distillation. The water/alcohol separation relies on the preferential affinity and diffusivity through the FO membrane of one of the compounds; however, studies remain relatively scarce and do not always show similar trends.

A first study evaluated the possibility for FO to concentrate ethanol (also called ethanol dehydration, in the feed solution) with draw recovery via solar energy through a theoretical approach. A concentration rate up to 50% ethanol purity seemed realistic, but recovery as high as 95% is not feasible due to high osmotic pressure required from the draw solution (e.g., 391 bar for 90% ethanol concentration) [67]. Zhang et al. investigated the use of FO for the concentration of bioethanol using a CTA membrane and highly concentrated NaCl solution as a draw (26%) and proved experimentally that ethanol content could be increased from 15 to 27.8% and from 82.5% to 90.1% thanks to the preferential permeation of water for the whole range of water/ethanol ratio tested. Even though promising, this process suffered from loss of ethanol (forward ethanol flux), reverse salt diffusion and degradation of the membrane after extended contact with concentrated ethanol solution [68]. Using a similar membrane, different results were obtained by Ambrosini et al. who demonstrated the possibility to preferentially removing alcohol from a feed solution (dealcoholisation). This study performed with lower initial alcohol concentration in the feed (5%) described how draw selection can impact relative alcohol/water passage through the membrane, organic draw solutes being preferred to favour alcohol permeation [69]. The same research team also investigated the impact of membrane type (CTA and TFC membranes from HTI) on the separation process [70]. The TFC membrane, which comparatively rejected better ethanol, proved to be less relevant in the context of dealcoholisation. Both alcohol dehydration and dealcoholisation and tunability to the application needs are promoted by FO membrane suppliers as part of FO concentration processes in alcoholic beverages [48,49,50]. However, a limited number of studies is available and, moreover, sometimes contradictory regarding preferential water and ethanol respective passage through FO membranes. Further work is required to clearly assess the interest and limitations of FO in this application both for alcohol concentration and dealcoholisation. The impact of initial alcohol/water ratio, type of draw solution and membrane type as well are more fundamentals of understanding of mass transfer are needed.

### 2.2. Organic Value-Added Compounds

#### 2.2.1. Food Derived Products and by-Products

Organic value-added compounds, such as proteins, peptides, antioxidants, colorants, flavours and dietary fibres can be recovered from food products or by-products (fruit, olive, dairy, cereals, seafood, slaughterhouse, etc.). Those products, also called nutraceuticals, can deliver health benefits on top of their nutritional value [71]. The recovery of those products relies on extraction (usually via solvent) and separation/concentration steps that can be performed through membrane processes. Initial tests with FO demonstrated the potential of this technology to concentrate twice a modelled solution of bovin serum albumin [72]. Then, several studies validated the concept on industrial by-products streams (Table 4): 9-times concentration of polyphenols from carob pulp was achieved as part of polyphenols process extraction and purification [73]; proteins and minerals from tuna cooking juice were also successfully concentrated by FO (from 4 to 9%) [74]; 2.15-times polyphenols concentration from olive mill WW [75]; 3- to 4-times melanoidins and antioxidants concentration from molasses distillery WW [76].

FO also proved to be of interest as a concentration process in the main production of anthocyanin extract from Garcinia indica Choisy (popularly known as kokum), concentrated 54 times (from 49 to 2.69 g·L^−1^) [45]. Sugar concentration is one of the targeted applications either for sugar juice production or downstream bioethanol production. Several studies confirmed the potential of either using xylose as a model solution [77] or real streams. A 4-fold concentration of sucrose from sugarcane juice (from 10.5 to 40.6%) [78] was achieved. In the context of sugar concentration for ethanol production from rice straw, FO proved to be beneficial in removing fermentation inhibitors and concentrating sugar from 199 up to 1612 mM and leading to improved production of ethanol [79,80]. A 5-fold concentration of fermentation products of commercial relevance (acetic acid, succinic acid, lactic acid and ethanol) was also achieved to facilitate their valorisation [81,82]. Other applications are envisioned in the protein and pharmaceutical industry [83]. Overall, most studies confirmed the high rejection of those organic compounds by FO membranes but also pointed out the need for adapted draw solution (not to pollute the feed stream) and indicated required pre-treatment and cleaning strategies to limit the fouling potential of certain molecules (such as pectins, for example).

**Table 4:** FO concentration studies on food derived products and by-products.

Application	Membrane Type	Draw Solution	Initial Jw (L·m^−2^·h^−1^)	Initial Conc.	CF	Final Conc.	Ref.
Polyphenols from carob pulp	CTA FO	K-lactate 60° Brix	13	1.13 g·L^−1^ TPC and 4.5°	16.2 (TPC)	18.4 g·L^−1^ TPC and 24.5°	[73]
Proteins/tuna cooking juice	CTA FO	0.5–2 M NaCl	5	5.5%	1.6	9.0%	[74]
Biophenols/olive mill WW	CTA FO	3 M MgCl_2_	6.5	>2 concentration on analysed phenolic			[75]
Polyphenols and melanoidins/Molassess distillery WW	TFC FO	4 M MgCl_2_	4	80 g·L^−1^ Mel, 10 g·L^−1^ Polyphenols	>2 concentration with 97% rejection of Melanoidins, 90% on antioxidant		[76]
Polyphenols and melanoidins/Molassess distillery WW	CTA & TFC	3 M MgCl_2_	4–6	80 g·L^−1^ Mel, 69 mM antioxydant	up to 4	141–267g·L^−1^ Mel, 102–213 mM antioxydant	[84]
Xylose	TFC FO	1–3 M MgCl_2_/LaCl_3_	25	25 g·L^−1^	6	150 g·L^−1^	[77]
Sugar cane juice/Sucrose	TFC FO	sea bittern (MgCl_2_)	13	10.50%	3.9	40.60%	[78]
Rice straw/Sugar	TFC FO	MgSO_4_ (saturated)		20 g·L^−1^	3.6	72 g·L^−1^	[79]
Pretreated-rice straw/Sugars	TFC FO	3.6 M TEA	4	199 mM	8.1	1612 mM	[80]
Fermentation broth/Succinic acid	TFC FO	5 M NaCl	16	40 g·L^−1^	4.5	180 g·L^−1^	[81]
Fermentation broth/Lactic acid	TFC FO	5 M NaCl	13	15 g·L^−1^	3.8	57 g·L^−1^	[81]
Fermentation broth/Ethanol	TFC FO	5 M NaCl	22	20 g·L^−1^	5.5	110 g·L^−1^	[81]

#### 2.2.2. Microalgae Harvesting

Microalgae are sunlight-driven cell factories that convert carbon dioxide to potential biofuels, foods, food supplements, feeds, fertilizer and high-value bioactive compounds [85,86,87]. Two main streams for microalgae production exist:(1) well controlled indoor cultivation systems in closed systems for high value product (food) and (2) lower cost open-air systems for less stringent applications. Along with the potential for CO_2_ sequestration, microalgae-based WW treatment is a promising approach to remove pollutants and produce biogas, biofuels and feeds [88,89]. Harvesting microalgae is a major challenge because of their small size, their density similar to water and their low concentration in the culture medium [90]. Currently, available processes involve two and more steps for concentration, including chemical or biological flocculation, centrifugation and/or filtration, as well as spray or thermal drying and extraction [91]. Utilizing a high energy intensive centrifuge system is economically justifiable only for high value-added product production [90]. The use of membrane technology in microalgae cultivation, harvesting and processing surged as a promising alternative, even though no commercial-scale application has been reported yet; hybrid processes (cultivation and separation) are very promising since they allow for process intensification [90].

Microalgae is effective to remove N and P and is generally envisioned as tertiary WW treatment; although using a bacteria-microalgae consortium also allows for chemical oxygen demand (COD) removal [92,93]. A recent review discussed applications of FO for microalgae which can be multiplied [94]; FO can be envisioned as a harvesting technology (concentration up to 1–5%). When compared to other membranes (UF/MF), the benefits of FO are the higher selectivity and nutrient removal rate from the water [95,96]. FO can be implemented as a standalone concentration step for concentration or within a hybrid, also known as osmotic membrane photobioreactor [97]. Among the FO initiative, the OMEGA Project evaluated the possibility to concentrate microalgae and harvest nutrients from WW using FO bags placed in seawater; concentration by 4- to 6.6-times were achieved in the lab and full scale demonstration was realised in a real environment [88,98].

Several bench scale studies using a cross flow setup confirmed the effective high removal of water and concentration of microalgae by FO; using the novel TFC membrane, Ye et al. achieved more than 20-times the concentration (from 1 up to 23 g·L^−1^) at high flux (above 20 L·m^−2^·h^−1^) and without major fouling issues [99]. Among others, the use of human urine and glycerol was successfully used as draw solution for microalgae concentration (by 4 and 2.5 folds, respectively) [100,101]. Recently, Ryu et al. demonstrated the feasibility to concentrate microalgae up to 120 g·L^−1^ using a glucose solution that is effectively diluted to be used as nutrients for microalgae, avoiding the need for a reconcentration process [102]. Still, effective permeation flux and concentration are largely dependent on the operation. Spacerless FO module feed channel design or submerged systems proved to limit biomass loss in the filtration system [99,103]. Biomass cultivating conditions and unexpected extrapolymeric substances production lead to enhanced fouling that is boosted by reverse salt diffusion (RSD) from the draw solution [103,104]. As for other FO applications especially dealing with difficult streams, draw selection and membrane orientation with active layer facing feed (AL-FS) are key elements in FO microalgae concentration process [99,104,105,106,107].

#### 2.2.3. Volatile Fatty Acid Production

An alternative method to the conversion of the organic fraction into energy as biogas is the production of volatile fatty acids (VFAs), which are the building blocks for a multitude of valuable products, such as biopolymers, medium, or long chain fatty acids and biofuels [108]. However, the generally low organic content of domestic WW (<300 mg L^−1^) hinders efficient recovery and is one of the main limitations in developing feasible bioproduction platforms. FO was evaluated as a pre-concentration step; it enabled the 10-fold dewatering of domestic WW, primary sludge and secondary activated sludge, but part of the COD was lost due to aeration, which promoted COD degradation [109]. Still, FO increased the soluble COD (sCOD) fraction in the sludge, enhancing VFA production by 7% for secondary sludge and by 35% for primary sludge (from 10% to 45%) but did not enable VFA production from the domestic WW due to the limited sCOD. FO concentration of VFA produced in a hydrolytic anaerobic reactor was studied to optimize the downstream microbial desalination cell (MDC) [82]. VFA rejection by the FO membrane was highly pH dependent [110]; at pH = 7.5 rejection rates above 80% are achievable and therefore the concentration of VFA by the FO process proved to be realistic and a 5-fold concentration from 60–80 up to 300–400 mg·L^−1^ was reached.

### 2.3. Water Reuse and Nutrients Recovery

**Figure 3:**
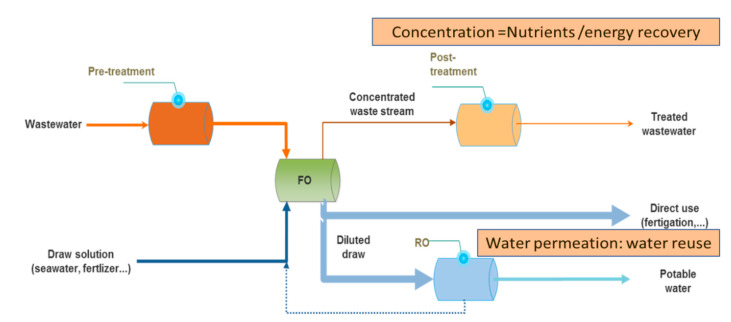
Integration of FO as a concentration process in the WW treatment line to combine water reuse and nutrients concentration.

The use of FO within the WW treatment line has been extensively studied in the last decade with the primary objective to promote water reuse; FO acts as a strong barrier that allows the purification and disinfection of water to be considered for this stringent application. Its implementation can be beneficial, especially if a saline stream is available (seawater, fertilizer, industrial brine, etc.) [111,112,113]. Using FO to combine water reuse and desalination (FO–RO hybrid) already demonstrated several advantages, such as double barrier protection (FO and RO both use dense membranes), and can have positive economics compared to stand-alone seawater RO desalination, due to energy (osmotic dilution) and maintenance savings, resulting from lower fouling tendency estimated from laboratory or pilot scale testing [114,115,116,117,118,119]. Recently, a first large scale pilot demonstration plant (1000 m^3^/day) was launched in Yeosu (South Korea) [120]. Initial studies have considered tertiary treated WW as a feed of the FO–RO hybrid. However, some recent lab-scale and pilot studies demonstrated that FO is a robust and simple process allowing us to treat even difficult streams, such as anaerobic digester concentrate or sludge [121,122]. Thus, instead of using tertiary or secondary treated WW, new concepts have emerged, including the implementation of FO upstream in the WW treatment scheme which can be beneficial not only in the context of water reuse but also for nutrients concentration to facilitate their downstream recovery (Figure 3 and Table 5).

#### 2.3.1. Primary Treated WW and Raw Sewage Concentration

FO treatment is envisioned on the raw WW after primary treatment or on raw sewage (sewer mining) [111,123]. The pre-concentration of low strength WW by FO when combined with anaerobic digestion, has several benefits, including higher biogas production, recovery of nutrients, lower FO membrane fouling propensity and smaller digester volume [111]. A large numbers of studies has been recently published relating the interests, limitations and optimisations of such schemes [124,125]. Several studies have already tried to implement FO for pre concentrating WW for subsequent treatment via anaerobic digestion. The first study by Zhang et al. achieved 6-fold concentration of primary treated WW using a lab-scale CTA cross-flow module, also leading to a 3.1 fold COD concentration [126]. Ansari et al. demonstrated the feasibility to concentrate, by 10-fold, WW, and to reach a 1000 mg·L^−1^ COD concentration, which is suitable for downstream anaerobic digestion [127]. Moving to a pilot scale, water concentration factors (WCF) of 5 and 2.5 were achieved with a spiral wound membrane and submerged plate and frame (P&F) TFC modules, respectively [128,129]. One of the limitations of raw WW concentration is the incomplete and heterogeneous recovery of nutrients (N, P, COD). COD is generally well rejected by the FO membranes (COD removal > 97%), but both studies showed that in-between 19.2% and 25.8% of the COD entering the system was lost due to biodegradation during the filtration process or attached to the membrane surface. While elevated phosphorous recovery was possible due to high rejection by the FO membranes, lower nitrogen recovery was observed, resulting from poor membrane rejection (up to 50–60%) [126,129,130].

Fouling is the main concern in membrane processes and especially when treating challenging feeds. Fouling was observed in most studies and especially when reaching high CF [127]. However, initial experiments using FO on primary treated (screened) WW demonstrated that the accumulated fouling layer was loose and easily reversible [111]. Additionally, the concentration of raw sewage using the submerged FO system proved to be promising with operation without clogging and no major fouling issue [128]. Another limitation of FO process concentration can be the toxicity of some compounds for downstream treatments—i.e., anaerobic digestion inhibitors, such as SO_4_^2−^, NH_3_, and salts. Apart from limiting the CF, organic draw solution (sodium acetate or EDTA-2Na) with lower RSD and biodegradability potential might be used [127]. Still, the filtration of WW and especially raw WW is as a major operating challenge and most studies were performed in lab or small scale pilots with very limited filtration time. Longer term assessments are required and are expected to reveal other practical issues and challenges.

#### 2.3.2. Osmotic Membrane Bioreactor (OMBR)

In OMBR [131], FO is implemented within the secondary (biological) treatment. Higher rejections of contaminants were observed than for the classical membrane bioreactor (MBR), yet at lower fouling propensity [132,133,134] and thus OMBRs can produce high water quality, which is crucial in the context of potable water reuse and rejection, and the degradation and/or concentration of most contaminants/nutrients, including trace organic contaminants [134,135]. One major limitation of OMBR is the salinity build-up impacting biological activity [136]. To tackle this issue, the addition of the UF or MF system into OMBR to create salt bleeding is promoted. This process is more difficult to operate since two sets of well-balanced membrane systems are needed [137,138] but can allow for the concentration of phosphorous to facilitate its downstream recovery [139,140]. Another configuration is the implementation of FO on the MBR permeate to avoid the salinity gradient in the biological reactor; Xue et al. demonstrated the possibility to operate such a system for phosphorous and nitrogen concentration achieving up to 4- and 2.1-fold concentrations, respectively, when concentrating WW four times. According to the modelling results, up to 10-fold of nutrient concentration could be achieved by optimising conditions for nitrogen rejection and using a ratio of seawater (draw):WW of 2:1 [141,142].

#### 2.3.3. Anaerobic OMBR (AnOMBR)

The anaerobic membrane bioreactor (AnMBR) combines anaerobic digestion technology with membrane technology. Unlike traditional anaerobic digestion, AnMBR allows for the treatment of low strength streams by decoupling the SRT from the HRT. In the last few years, many studies focused on the direct treatment of municipal WW via AnMBR at lab and pilot-scale [143], but the low methane production and the energy needed for membrane operation (biogas sparging and permeate pump) and for maintaining the reactor temperature limited its broader development [144]. Combining FO with AnMBR has developed an increasing interest to increase COD load and improve biogas production. These technologies can be combined in two different ways: (1) by replacing or coupling the MF or UF membrane system with an FO system in an AnOMBR system or (2) by using FO to pre-concentrate WW for subsequent anaerobic treatment.

The operation of AnOMBR positively led to almost total removal of COD and phosphorous and 62% removal of ammonium-nitrogen which is a bit higher than conventional AnMBR. Additionally, 0.21 L·CH_4_·g·COD^−1^ of biogas was consistently produced even after salinity build up that did not seem to impact biological activity [145]. Similar observations than for OMBR were drawn—i.e., high rejection rates, moderate fouling, but severe salinity build-up overtime when only an FO membrane is used [146]. Even if in some cases, biogas production did not seem impacted by salinity build-up, Tang et al. observed that it negatively affected methanogenic growth, leading to the ousting of methanogens by sulphate-reducing bacteria [147]. As for OMBR, combining the FO and MF membranes into the AnMBR reactor was also evaluated and positively avoided salinity build-up while assuring the production of high water quality (through the FO membrane), production of biogas and concentration of nutrients (phosphorous in the MF permeate) to facilitate its precipitation [148].

The water production cost associated with the combination of FO-RO-AnMBR for municipal WW treatment was estimated by Vinardell et al. [149] for four different scenarios, AnMBR directly treating WW and FO-RO-AnMBR with different FO water recoveries (50, 80, 90%). WW treatment cost below 1 €·m^−3^ was achieved when FO recovery was limited to 50%; increasing FO flux above 10 L·m^−2^·h^−1^ was identified as a key factor to improve significantly the competitiveness of the FO–RO–AnMBR system.

**Table 5:** Overview of FO integration in WW processes and efficiency in rejection or concentration of nutrients. TOC, COD, NH4, TN and TP expressed in % removal or concentration factor (CF). (sub-FO: submerged forward osmosis, P&F: Plate and frame module)

Process	Membrane Type	Draw Solution	TOC Removal (%) or CF	COD Removal (%) or CF	NH4 Removal (%) or CF	TN Removal (%) or CF	TP Removal (%) or CF	Ref.
OMBR	CTA (cross-flow)	NaCl 1 M	98%	-	99%	-	-	[150]
	TFC (cross-flow)	NaCl 1 M	96%	-	99%	-	-	[150]
	CTA (sub-FO P&F)	NaCl 0.7 M	-	>99%	-	>82%	>99%	[151]
	CTA (sub-FO P&F)	NaCl and MgCl_2_	98%	-	80–90%	-	>99% (P-PO_4_)	[152]
	CTA (sub-FO P&F)	NaCl	>98%	-	>98%	-	-	[153]
	CTA (sub-FO P&F)	-	-	>95%	70–80%	-	>99%	[154]
An-OMBR	CTA (sub-FO P&F)	-	-	>95%	70–80%	-	>99%	[146]
	CTA (sub-FO P&F)	NaCl	-	96.7%	60%	-	99%	[145]
	TFC (Aquaporin)	MgSO4	-	>95%	>95%	-	>95%	[155]
	CTA (sub-FO P&F)	NaCl and Na_2_SO_4_	92.9%	-		-	-	[148]
WW pre conc.	CTA (sub-FO P&F)	-	-	99%	-	56–59%	99% (P-PO_4_)	[113]
	CTA (pilot-SW)	0.5 M NaCl	-	99.8%	48.1%	67.8%	99.7%	[129]
	TFC	Synthetic seawater brine	-	CF 2.4 and 2.7	CF 1.3 and 1.8	CF 1.6 and 1.9	CF 3.3 and 3.5	[156]
	CTA	Synthetic seawater	79.0 ± 6.7%	-	85.4%	-	93.3 ± 3.3%	[154]
	CTA-ES	0.2–4 M NaCl	-	96.5%	89.4%	93.3%	95.4%	[157]
	TFC-Aquaporin	1.5 M MgCl_2_	-	99%	CF 1.32	-	68–74%	[158]
	TFC	-	-	93.0–97.5	<59.06%	-	93.7–98.5% (P-PO_4_)	[130]
	CTA	1 M NaCl	-	97%	-	96%	>99%	[159]
	TFC Aquaporin	1 M NaCl	-	97%	-	96%	>99%	[159]
	CTA	0.6 M NaCl	98%	-	70%	-	90%	[160]
	TFC (sub-FO P&F)	0.2 M Seasalts	-	CF 2.4	CF 0.75	-	CF 2.2	[128]
	CTA	Synthetic seawater	-	80–95%	89.2%	-	99%	[161]

### 2.4. Treatment of Waste Streams

#### 2.4.1. Sludge Concentration

Sludge produced during the purification processes of WW generally needs to be concentrated through successive steps, such as thickening, dewatering and possibly drying and stabilisation steps, such as anaerobic digestion or liming before reuse or disposal. Those steps are energetically intensive and require chemicals; the opportunity to use osmotic energy for their concentration may be of significant economic interest.

When FO was applied on aerated biological sludge, up to 7-fold concentration of MLSS was achieved (from 3 to 21 g·L^−1^) and with a high retention rate (>96%) of nitrogen and phosphorous, allowing for the production of high-nutrient sludge [122]. The potential of FO as a thickening step was also demonstrated by Zhu et al., where MLSS could be increased from 7 to 39 g·L^−1^ so to favour downstream anaerobic digestion efficiency [162]. To limit RSD occurring with NaCl or seawater as a draw solution, several studies investigated concentration with alternative draws. Na_3_PO_4_ proved to be a suitable draw agent, allowing for sludge concentration from 3.5 up to 22 g·L^−1^ with 100% phosphate rejection and concentration from the feed and low RSD (0.07g·L^−1^) [163]. Similar trends were observed using Ethylenediaminetetraacetic acid (EDTA) as a draw with high rejections of feed nutrients and concentration of sludge from 8 up to 32 g·L^−1^ [164].

Following the thickening step, aerated sludge at 7 g·L^−1^ was further concentrated up to 35% dry solids content using the thin bed dewatering FO system [162]. Most studies observed cake-layer formation and fouling propensity given the concentration rate of suspended solids achieved. However, in most cases fouling was easily reversible even in the case of minerals precipitation. Still, antifouling strategies should be envisioned especially in the case of sludge dewatering where cake formation is inherent to the process. The application of ultrasounds for fouling mitigation proved to impact sludge flocs; mitigation of fouling deposition was observed only under mild/controlled conditions—continuous ultrasound radiation applications may cause high organic release from sludge flocs, thus leading to higher fouling rate [165,166]. Module scale testing considering adapted module design to tackle clogging, and high solid concentration issues and anti-fouling strategies are still to be evaluated to further assess the potential of FO for sludge concentration.

#### 2.4.2. Urine Recovery and Concentration

Despite its low volumetric load (less than 1% of the WW volume), this accounts for approximately 80% of the nitrogen, 50% of the phosphorus and 55% of the potassium load in most of the WW treatment plants [167]. Separating urine from domestic WW promotes a more sustainable municipal WW treatment system [168]. Moreover, phosphorus and nitrogen contents could be mined; human urine has often proven to be a suitable raw material for fertiliser production. However, most of the urine-diverting toilets or male urinals result in a dilution of 2- to 10-times, which decreases its efficiency for further recovery and reuse [169]. Several physicochemical and biological processes have been developed for nutrient recovery and/or removal from urine, such as struvite precipitation, ammonia stripping, and the nitrification−denitrification process, and they generally require high energy, chemical and investment costs [170].

Several recent studies demonstrated the potential of FO as a concentration/separation process for ammonium and phosphorous recovery from source separated urine. The feasibility of urine concentration by FO was demonstrated, with up to a 5-fold concentration using a 5 M NaCl solution [171]. If phosphorous and potassium are concentrated, one main challenge is the relatively poor rejection of nitrogen especially under urea form by FO membranes (50–60%) [171]. In that context, the improved rejection of nitrogen (50−80%) in hydrolysed urine was observed in comparison with the low rejection of neutral organic nitrogen (urea-N) in fresh urine [168]. A pH adjustment of around 7 also favoured the higher rejection of ammonium (around 80%) [172] by avoiding the stripping of ammonia occurring at higher natural pH of urine (above 8) and limiting potential membrane scaling [173]. The concept has been integrated within a FO-MD hybrid for the simultaneous production of fresh water and urine concentration [173,174,175]. Given the moderate rejection of urea by the FO membrane, Volpin et al. combined FO concentration processes with fertigation using a Mg based fertiliser as a draw solution. In such a scheme, urine passing through the membrane enriches the fertilizer with ammonium, while phosphorous and the remaining nitrogen and magnesium are concentrated on the feed side and can be recovered as struvite precipitate [169,176]. The revenue of such a scheme being potentially more than 5-times the associated (operational and capital) costs.

#### 2.4.3. Landfill Leachate

Thanks to its low exploitation and capital costs, landfilling is a widely used process for disposing of municipal solid waste. Landfill implies the generation of leachate, a highly polluted WW that must be treated prior to discharge [177]. Landfill leachate typically contains high amounts of organic matter, ammonium, heavy metals and chlorinated organic and inorganic salts, rending its treatment complex and expensive. Several approaches mention FO as a concentration step of the treatment train to lower costs and enhance the recovery of nutrients and water [178]. Long term pilot testing demonstrated that the FO system (coupled with RO for draw recovery) could concentrate, by 10-fold, landfill leachate before further crystallisation and solidification with cement, which was returned to the landfill [179]. By concentrating by 3.2 folds through FO, Iskander et al. observed that, by incorporating FO and humic acid recovery into a Fenton’s oxidation system, FO could decrease leachate volume, lower reagent requirements, and reduce sludge production by 30% [180]. Even at a moderate concentration rate (1.5 fold), and after calcium pre-treatment, FO was used to successfully precipitate struvite [181]. FO, combined with microbial desalination cells also proved to be feasible to concentrate leachate and recover nitrogen [182,183]. Several other studies demonstrated the positive impact of FO as part of the leachate treatment line to produce fresh water and remove contaminants within the FO–MD hybrid, in FO-fertigation or as a post-treatment of the MBR permeate, even though the CF in FO is not generally mentioned [184,185,186].

#### 2.4.4. Digestate Treatment

Digestate, in some cases, can be applied onto farmlands to enhance crop yields, as it is abundant in nutrients (COD, N, and P) [187]. However, in some areas, digestate production exceeds the carrying capacity of lands nearby or is sometimes polluted. Thus, technologies focused on concentration and/or nutrient recovery from digestate have been studied recently [187,188]. Digestate from various sources (potato starch WW, swine manure and two types of cattle manure) were comparatively tested for concentration to enhance anaerobic digestion (side stream FO-AnMBR); 1.3-fold CF allows for the significant enhancement of biogas production [189]. Other studies evaluated the possibility to reject and concentrate organic fraction, N and P from manure digestate using FO. Even if manure CF was limited to two in most cases, the effective concentration of nutrients was achieved. P rejection was high in all cases (>95%) but the very variable rejection of nitrogen was observed from less than 40% in some cases and up to nearly total rejection without clear explanation [190,191,192,193]. P can be recovered as struvite if precipitation is controlled; otherwise, it may lead to a scaling issue [192,194]. Li et al. mentioned that FO implementation prior to struvite precipitation with MgCl_2_ showed superior performance in P recovery and can generate a value of 1.35 $·m^−3^, thanks to recovered struvite and water [191]. The possibility to recover phosphate from sludge digestate concentrate was also successfully achieved by concentrating the streams up to the limit of solubility so as to precipitate phosphorous as calcium phosphate [195] or struvite (using MgCl_2_ as draw) [30]. Fouling propensity was moderate in all cases, even though it can be explained by low permeation flux and by limited time of operation (few hours) and at lab-scale. The fate of heavy metals and antibiotics from manure was also evaluated [191]; heavy metals were highly rejected by FO membranes while antibiotics are more membrane- and compound-dependent. Overall, both antibiotics and heavy metals tend to accumulate in the concentrated stream and should be considered for downstream treatment and applications.

### 2.5. Brine Management: Concentration and Salts Mining

#### 2.5.1. Brine Concentration Towards Zero Liquid Discharge

Brines are produced by water desalination system (concentrate from RO of brackish and seawater desalination plant) and from various industrial sectors (mining, pulp and paper, food industry, chemicals, etc.). The proper disposal and management of brines, and their highly concentrated salts, organics, and other contaminants, represent a significant environmental challenge to most plants [196]. RO brines, especially inland brines, still require appropriate management solutions [197]. Conventional disposal options for concentrate include surface water discharge, discharge to the WW treatment plant, deep well injection, evaporation ponds and land application [198]. However, these brine disposal methods are unsustainable and restricted by high capital costs. Nowadays, brine treatment is heading towards a zero-liquid discharge (ZLD) approach, providing water reuse and waste volume minimization [199]. Among other innovative processes, FO is gaining attention for brine concentration. Among the few academic studies found [200,201,202], concentration from 73 up to 180 g·L^−1^ via an NH_3_/CO_2_ FO membrane brine concentrator (MBC) was achieved on produced waters from natural gas extraction operations [202]. This process allowed for higher concentration than high pressure RO and 42% less energy, which is greater than the mechanical vapor compression evaporator. In total, 90% water extraction from brackish water brine was also obtained using FO, higher than MD for similar stream [201]. Full competitiveness versus existing concentration processes is still to be demonstrated on large scale and ways of improvement remain, especially with regard to flowrates and module design [203]. Despite relatively little literature, FO-based brine treatments towards ZLD systems are now commercialised [204,205,206].

#### 2.5.2. Seawater and Brine Mining/ Recovery of Valuable Minerals

Given the continuously increasing issues of land-based mining industries with regard to the depletion of high-grade ores, important water and energy demand and environmental issues, seawater and brines, containing large quantities of valuable minerals mining, have become an attractive alternative option to harvest minerals [207]. Apart from salt (NaCl), several minerals, such as Na_2_SO_4_, Li and Mg have been identified as specific targets and as economically attractive [207,208]. The main methods identified for the recovery of minerals are solar evaporation, electrodialysis, membrane distillation, crystallization, adsorption/desorption; however, novel membranes technologies including osmotic processes such as pressure retarded osmosis or FO potentially have a role to play towards extraction and concentration of minerals [208,209]. Scientific studies remain limited and focused on salt lake brines; the possibility to concentrate Li (and Mg) by 2.8-fold (from 0.78 to 2.8 g·L^−1^) [210] and to integrate FO and crystallisation for precipitation as lithium carbonate were demonstrated [211].

Van Wyk reviewed the state of the art and conducted experiments to better assess Lithium and boron rejection and associated mechanisms [212]. In opposition to TFC, the RO membrane, which can feature typically 90% boron rejection [213], studies conducted in FO reported lower and very variable rejection (from 10 to 100%). Boron rejection is generally higher using active layer facing feed solution (AL-FS) membrane orientation (higher electrostatic rejection), can be improved by dedicated membranes and increase at higher pH when boric acid is converted in borate anions which have a higher hydrated radius, which favours steric rejection [214,215,216,217,218]. Van Wyk confirmed the possibility to reach high rejection of boron (>90%) using the TFC membrane in AL-FS mode even at neutral pH [212]. Lithium rejection was first assessed by Coday et al., who observed a 78–88% efficiency with a TFC FO membrane [219]. This range of value was confirmed by Van Wyk who observed higher rejection of lithium when operating in an active-layer-facing draw solution (AL-DS) membrane orientation and concluded that lithium rejection was driven by electrostatic interactions and steric rejection [212].

## 3. Tuning FO Process to Tackle Current Limitations for Optimum Concentration

### 3.1. Better Understanding of FO Fundamentals in the Context of Concentration

The performances of FO concentration processes are sometimes difficult to assess and compare due to the lack of uniformed terminology and systematic methodology. Some studies refer to rejection, others to concentration of feed component or % of water recovery. Overall, concentrations of compounds from the feed depends on the concentration of water and then on their fate during the concentration process; phenomena such as partial rejection by the membrane, adsorption, biological degradation, deposition of precipitation may happen during the concentration by FO, influencing the concentration of those compounds. The following section aims first at proposing a methodology to properly assess the concentration of water and nutrients/contaminants in FO process. At first, WCF as a key element for the evaluation for effective concentration will be introduced. Then, current knowledge of rejection of both mineral and organic elements by FO membranes will be reported as well as potential effects, such as biodegradation and deposition/clogging that may affect effective recovery.

#### 3.1.1. Water Concentration Factor (WCF)

The WCF represents the concentration of the feed solution at a given time and is calculated using Equation (1):

(1) $$\mathrm{WCF}\;=\frac{V_{Fi}}{V_{F}\left(t\right)}$$

where *V_Fi_* is the feed initial volume and *V_F_*(t) the feed volume at any moment (t) during the filtration.

Of specific interest is the achievable WCF (WCF_a_), which corresponds to the theoretically achievable WCF when osmotic equilibrium is reached. Such a factor is of specific interest to design the concentration process and is a function of feed and draw solutions initial osmotic pressures and volumes. Assuming that the membrane is only permeable to water, Equation (2) is used to calculate the osmotic pressure at equilibrium, *π_equ_*:

(2) $$\pi_{equ}=\frac{(V_{Fi}*\pi_{Fi})+\left(V_{Di}*\pi_{Di}\right)}{V_{Fi}+V_{Di}}$$

where *π_Fi_*, *π_Di_* and *V_Di_* are initial osmotic pressure of the feed solution, initial osmotic pressure of the draw solution and initial volume of the draw solution, respectively. Assuming there is no loss of salts, *V_Fequ_*, the volume of the feed solution at the equilibrium can be calculated using Equation (3):

(3) $$V_{Fequ}*\pi_{equ}=V_{Fi}*\pi_{Fi}$$

WCF_a_ can be then calculated at the equilibrium by using *V_Fequ_* as a value of *V_F_*(t) at the equilibirum:

(4) $${\mathrm{WCF}}_{a}\;=\frac{1}{\pi_{Fi}}*\frac{(V_{Fi}*\pi_{Fi})+\left(V_{Di}*\pi_{Di}\right)}{V_{Fi}*V_{Di}}$$

**Figure 4:**
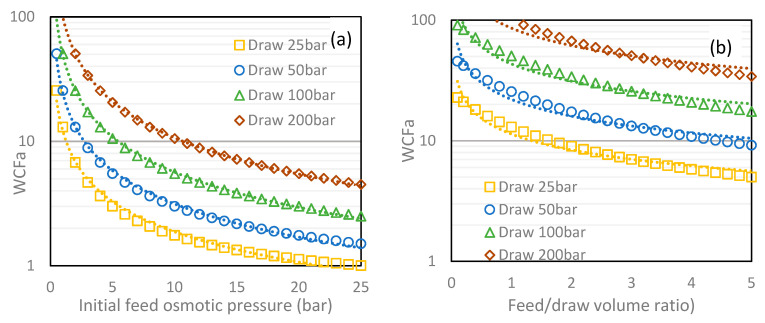
Modelling of WCF_a_ as function of (**a**) draw solute concentration and initial feed osmotic pressure (feed draw ratio 1), (**b**) feed draw ratio and draw osmotic pressure for *π_F_* = 1 bar.

Based on Equation (4), estimations on WCF by FO can be approximated. In Figure 4 are reported various configurations and the impact of operating conditions on the theoretically achievable WCF using batch operation. The impact of initial feed concentration, initial draw concentration, ratio feed/draw volume as well as the imperfect rejection of the draw solution by the membrane (RSD) are evaluated. It is important to understand that such model do not consider filtration kinetics or module design and only rely on batch operation (i.e., defined volumes and concentrations of feed and draw are implemented and the WCF_a_ is achieved when osmotic equilibrium after indefinite time is reached in between the two solutions).

As observed in Figure 4, WCFa higher than 100 can theoretically be achieved. Obviously, the highest WCF_a_ corresponds to the conditions where initial feed concentration is minimal, initial draw solution concentration is the highest, and feed-to-draw ratio is minimal. In practice, a WCF higher than 100 is rarely aimed and could be limited by other process limitations, such as scaling, viscosity, or very long kinetics, especially when approaching the osmotic equilibrium. A 10-fold concentration rate is achieved for a broad range of operating conditions and is a more realistic target for FO as a concentration process. Refined models need to consider RSD and ideally also forward solute flux (FSD), which are key parameters affecting the WCF_a_, since any leakage of solutes will decrease osmotic gradient and shift the osmotic equilibrium. It is expected that, at low WCF, RSD has a minimal impact but becomes critical when WCF > 10 is expected.

#### 3.1.2. Nutrients Rejections and Recovery

Large molecules, such as proteins, (typically above 1000 kDa) are highly rejected by FO membranes and high CF are achieved. Pectins and humic acids feature very high molecular weight; tannins and polyphenols are smaller molecules (500–4000 Da) but are still well above the cut-off of FO membranes. However, imperfect rejection by the FO membranes of small organics, such as VFA, alcohols, pharmaceuticals and ions (cations especially, ammonium and nitrate more specifically) has been reported [25,110,220,221]. Overall, if the predominance of steric rejection is assumed for large molecules, other mechanisms may become preponderant for small ones, typically for molecular weight below 400 Da. Specific rejection studies regarding low MW organics by FO were limited. Even if, in general, rejection remains high, some compounds were poorly rejected (sometimes below 50%) under specific operating conditions (pH, membrane and draw type) especially for compounds below 100 Da (ethanol, acetic acid, urea, phenol) or some uncharged micropollutants even larger than 200 Da [82,110,212,222,223,224].

Electrostatic interactions between the membrane and feed solutes are also to be considered. While CTA membranes have a negligible charge, the TFC membrane surface features carboxyl groups more negatively charged, which could serve as a fixed ionic group, thereby conferring to the membrane a cation exchange feature [225]. Several studies demonstrated that negatively charged membranes better rejected negatively charged compounds/anions while positively charged/cations were more poorly rejected [222,226,227]. Using the TFC membrane and NaCl as draw, Ferrari et al. observed a very strong effect of cations/anions selectivity. Most cations present in the WW were poorly rejected (NH_4_^+^, K^+^, Na^+^, Ca^2+^), while anions (SO_4_^2−^, PO_4_^3−^) were effectively concentrated [128]. Additionally, bidirectional solute flux plays a role [226,228,229]. When an ion with high diffusivity passes through the FO membrane, the system tries to maintain solution electroneutrality by transporting the less diffusive counter-anion; the low diffusing ion being the limiting one for the diffusion of both counter ions [229,230]. A similar phenomenon occurred for both directions. Some electrostatic interactions may occur between feed and draw ions—i.e., a draw electrolyte composed of a low-diffusivity cation and high-diffusivity anion may promote the forward transport of feed anions and retarded that of feed cations. Conversely, the forward transport of feed anions may be greatly reduced, while that of the feed cation significantly enhanced. Such a phenomenon, also referred to as Donnan equilibrium, was evidenced especially for the rejection of nitrate and ammonium, draw selection having a great impact in favouring or retarding their forward diffusion [221,226,228]. In addition to Donnan equilibrium, D´haese mentioned frictional phenomena, also called draw steric hindrance by Xie et al. as a potential mechanism impacting forward solute flux [231,232].

pH of the solution to be concentrated does have a role in ion transport for TFC membranes. As explained in the study by Lu et al., when the pH of the solution facing the active layer increases from 3 to 6, protonated carboxylic groups are substituted by deprotonated carboxylic groups, which make the negatively charged membrane surface influence electrostatic interactions [226]. A couple of studies related to VFA rejection by FO also demonstrated a strong pH dependence of the rejection and on their effective concentration. At pH 4, below VFA’s pKA, rejection relies solely on steric rejection, while at pH above 6, VFA exists mostly as negatively charged ions (R–COO–); electrostatic repulsion with the negatively charged membranes significantly enhanced VFA rejection [82,110]. In the case of uncharged compounds (no electrostatic interactions), hydrophilicity/hydrophobicity may have a role in organic compound rejection. It was demonstrated that hydrophobic compounds may be adsorbed on the hydrophobic membrane surface. However, high rejections due to adsorption are typically only a temporarily effect, until membrane saturation occurs [233]; then adsorbed molecules may be transported to a higher extent due to their higher partition coefficient [234]. Reversely, D’haese demonstrated that small hydrophilic organics (such as alcohols) may show negative rejection (up to 4–5 times concentration in the permeate), their transfer being promoted by salting out [220].

While FO systems treating WW have always demonstrated high COD and PO_4_^3−^ rejection (Table 5), diverse levels of cations rejection are observed. Among cations, NH_4_^+^ rejection is of major concern as it is of interest for many applications connected to the recovery of water and nutrients from waste streams. Poor NH_4_^+^ selectivity by TFC membranes is of major concern due to the extremely similar polarity and hydraulic radius between NH_4_^+^ and water molecules [125]. Nitrogen recovery by FO suffers from imperfect rejection not only in NH_4_^+^ form but also in NO_3_^−^ or urea, demonstrating that the electrostatic effect is not the only rejection mechanism in that case [128,174,221].

Apart from the imperfect rejection by FO membranes, deposition or biodegration may alter the effective concentration of organic compounds. Ferrari et al. indicated that up to 15% of COD was lost during concentration of raw WW in the submerged FO reactor [128]. The natural degradation of COD during filtration (20 h in that case) was found to be the most probable cause. In the context of VFA concentration, Blandin et al. [82] also observed a limited recovery (70–80%) of acetic acid due to biodegradation over filtration time. Long filtration time, the formation of a biofouling layer consuming highly biodegradable VFA and contact with air were found to be all factors affecting VFA imperfect recovery. The implementation of pretreatment to limit biofouling and periodic cleaning were applied to limit VFA biodegration. Onyshchenko et al. also reported the ineffective concentration of microalgae due to a deposition in membrane spacers during FO filtration [95]. Using spacer-less channel or submerged operation were proposed as improved design to limit deposition [102,103].

Very importantly, the reduction in the rejection with increasing recovery was observed, especially for compounds, such as urea, that were imperfectly rejected by FO membranes. This was expected since high recovery results in an increase in the solute concentration in the module [224]. Such observation is key for concentration process and reinforces the need for a high rejecting FO system. Further studies are required to assess the specific rejection of organic compounds to be recovered both at a fundamental level to understand mechanisms for rejection and using real feed solution that may consider complexations of those compounds.

### 3.2. Membranes and Modules

The parameters used to characterize these membranes are typically the pure water and salt permeability of the rejection layer (factors A and B, respectively), and the structural parameter of the support layer (S). The ideal FO membrane features a high A value (high water flux), low B (low salt passage), low S (to limitICP) and sufficient mechanical strength to support industrial operation at moderate pressure [17]. Huge membrane development was and still is conducted regarding novel membranes, focusing both on the membrane active and support layers to improve permeability, selectivity and limit internal ICP [23]. Overall, the findings from academic research have been translated into the development and commercialization of several FO membranes with improved flux and selectivity (up to 30 L·m^−2^·h^−1^ and with RSD below 0.5 g·L^−1^ when using 1 M NaCl draw solutions). Further improvements are desired, but developing membranes with enhanced selectivity, rather than higher flux, are required, especially for poorly rejected compounds, such as NH_4_^+^. In the context of NH_4_^+^ recovery, modifying the active layer with polyethylenimine (PEI), due to its abundant positively charged amines, allows an increase of 15–25% in the NH_4_^+^ removal efficiency [235]. High NH_4_^+^ rejections of 97%, comparable to CTA membranes [190] were also observed with the TFC membrane enriched with Aquaporin selective water channel proteins, which only allow water to pass through and reject ions.

Among the challenges to overcome in FO, module design is certainly of high importance. An ideal FO module is a trade-off between (1) a maximised surface area (i.e., high packing density), (2) a minimised pressure drop and (3) limited ECP and particle deposition [31,117]. Three different designs are currently commercial—i.e., P&F, hollow fibre (HF) and spiral-wound (SW) module configurations [236,237,238,239,240]. Tubular membrane configuration is under development but not commercialised yet [241]. Among the challenges of cross flow module scale operation, especially in treating complex or concentrated stream, is the necessity to transfer the feed solution through the modules and the associated fouling/clogging issues. In order to be optimised for challenging complex stream, SW modules feed spacer type can be adapted, P&F may be operated without a spacer and HF/tubular membranes inner fibre diameter can be adjusted. Still, deposition can occur, and pre-treatment may be required. Another option is the development of submerged configurations, as for MBR, where the FO membranes are immersed in the feed solution and are less affected by clogging [128,242]. Additionally, this configuration, owing to its limited shear stress for applications, may be of interest for cultivation of bioproducts, and concentrations to avoid the breakage of fragile biological compounds [243].

Both experimental and computational fluid dynamics, working at the module scale, demonstrated the need to improve the understanding of process limitations that could be affected both by channel design and pressure balance in FO modules [237,244,245,246,247,248,249,250,251,252]. Further studies are required with specific focus on the concentration process with regard to practical operation and limitations.

### 3.3. Process Design

When comparing FO with other membrane filtration processes, one key element is the absence of hydraulic pressure for water permeation that has important implications in terms of process design. First, operating at low pressure does not require mechanically resistant materials, as in NF/RO, and therefore the associated CAPEX costs are limited. Moreover, it allows for more flexibility on the process and module design and arrangements [253]. In FO, in opposition to NF/RO, typically limited to spiral wound cross flow modules, several other module designs can be envisioned, such as plate and frame, hollow fibre, tubular and submerged modules. The main implication, especially for tubular and submerged modules, is the opportunity to treat complex streams without major risk of clogging or extended pre-treatment required [128]. Another implication is that pressurisation of the feed solution, which is a large part of energy consumption and overall operation costs in NF/RO, is not a limiting factor for process design. Thus, one pass through modules in continuous mode is not necessary. The FO system could be operated either in batch or continuous mode. Designing osmotic processes with multi-stage and various draw-driving forces during the concentration process is also more flexible than when done with hydraulic pressure. While the FO process was initially operated at low flux, due to the first generation of membrane performances, novel TFC membranes suffer less from low flux comparison with regard to other membrane technologies. Additionally, since operation at higher flux led to more fouling propensity, recent studies on FO technologies at the module(s) scale identified several recommendations for its optimum integration with the objective to operate at constant driving force (and flux), rather than providing concentrated draw solution at the beginning of the FO process where the feed solution has the lowest salinity and therefore the osmotic driving force is the highest.

**Figure 5:**
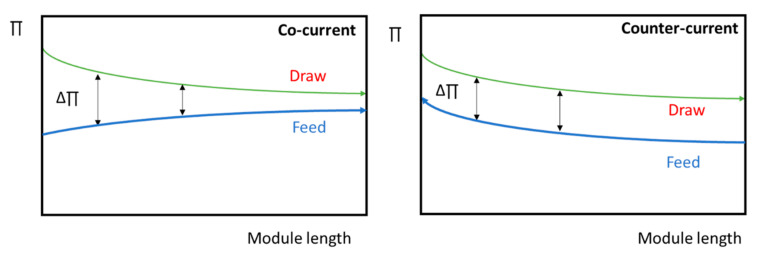
Description of osmotic pressure of feed and draw stream in along a FO module, depending on operation in co- and counter-current (adapted from [224]).

One recommendation is to operate FO in counter current mode to maintain a more constant osmotic pressure over the module(s) (Figure 5; [224]). Such an approach of operating offers the double benefit of (i) avoiding elevated flux at the start of the concentration process, limiting the fouling propensity and (ii) to still maintain a flux at the end of the concentration process instead of having difficulties when reaching the osmotic equilibrium [248,249]. Phuntsho et al. demonstrated that operating in counter-current allows for a higher average permeation flux and water extraction rate for the same filtration area [250,254]. Even more interesting is the fact that this mode of operation allows us to shift the osmotic equilibrium to increase the extraction capacity of a draw solutes and therefore the concentration capacity of the feed stream [254]. As a final step for concentration, pressure assisted osmosis may be used to overcome osmotic equilibrium limitations and reach a higher concentration level if required [255].

If FO concentration may be considered as a one step process (even under multiple stage operation), at a lab scale (lab scale/pilots) or even for some applications, the recirculation of the concentrated feed stream may be preferred. In that case, the concentrated feed coming out of the FO system is mixed with the inlet feed stream and concentration occurs progressively with feed volume, constantly decreasing in the feed tank. If high CF is aimed, the process design should consider the feed tank and piping, allowing us to operate with a broad range of volume (ideally conic tank, limiting piping volume, adapted sensors). In existing studies (lab, pilot scale), the draw side is either operated similarly in batch (with decreasing osmotic pressure occurring with dilution) or at a constant draw osmotic pressure (reconcentration by topping up with a highly concentrated draw solution or using a reconcentrating draw solution process (RO, MD, etc.)). One could also operate with increased draw concentration over the concentration process at a lab scale level, mimicking the counter-current continuous operation of a full-scale process.

### 3.4. Draw Selection

Taking advantage of the osmotic gradient, FO on its own can be considered as a low energy process and only requires limited energy for stream circulation within FO modules. However, pure water extracted from the feed solution is only transferred to a (draw) solution with a higher osmotic potential, and as such, is rarely usable as is—applications in which FO can be used as a stand-alone process without draw regeneration are limited [117]. The draw regeneration requires the use of a second process, which is generally energy intensive (RO/NF/MD, thermal process, etc.). An ideal draw solution must have the following characteristics: allowing for high water flux, minimal reverse draw solute flux, no toxicity, reasonably low cost, easy and low-cost recovery and compatible with feed purpose (facing the draw salt diffusion risk). Numerous studies reported various draw solutions and draw recovery processes (Table 6; [26,256,257]). Draw selection in the context of concentration will involve the specific consideration of the following elements:

- Osmotic pressure should be in line with the required concentration level and the initial osmotic pressure of the feed solution to be treated. Required osmotic pressure will not only impact the draw selection but also the draw recovery method, since above 60 bar typical RO recovery is limited and thermal processes, such as MD, evaporation processes, or under development thermo-responsive draw solution processes are required [258]. According to Siew, the RO draw regeneration system can be used up to 50–60 bar draw osmotic pressure with a range of energy consumption between 1 and 4 Kwh·m^−3^. From 40 to 120 bar, the RO system can be operated in osmotically assisted RO mode but requires high energy input (5–15 Kwh·m^−3^). As alternatives, MD, evaporators or thermo-responsive systems allow for a much broader range of operation—i.e., even as high as 200 Bar of draw osmotic pressure but with very high energy requirements.
- Compatibility with the solution to be treated is critical, especially for food and beverages or potable water reuse applications. The non-food draw solution may simply not be approved. Additionally, RSD may alter taste or other organoleptic properties. RSD can also be a limiting factor with regard to achieving high recovery (by shifting the osmotic equilibrium) or may alter biological activity (by excess of saline compounds).
- Costs are very application-dependent. If in food and beverages or brine concentration, more flexibility in draw selection and recovery may be allowed thanks to the relative low cost of FO vs. other concentration process. In lower costs applications, such as in WW treatment, a low cost solution will be preferred [259,260].

### 3.5. Fouling, Mitigation and Cleaning

Fouling mechanisms in osmotic processes were extensively reviewed by She et al. [24]. Lower fouling behaviour, commonly accepted in FO in comparison with the pressure driven system is currently a hot topic of debate [114,261,262,263,264]. It was demonstrated that low fouling behaviour in early studies was the consequence of operating at a low permeation flux (below 10 L·m^−2^·h^−1^). More severe fouling is observed when operating at a higher flux, also demonstrating the existence of a critical flux in FO [134,265,266]. This has important implications for FO competitive advantages and optimized operation, especially for concentration purposes where highly concentrated streams are processed. Designing systems with moderate but constant osmotic driving forces are preferred to systems with initial high osmotic gradients. Membrane cleaning techniques are similar to those employed for RO and NF for cross flow modules using physical cleaning by flushing [267], chemical cleaning [268,269] or those of MBR, such as air/gas scouring and vibrations for submerged FO processes [242,270,271]. Specific to FO, osmotic backwashing (similar to hydraulic backwashing in pressure-driven membranes) proved to be the most promising technique, especially used in combination with flushing by detaching the fouling layer from the membranes and facilitating its diffusion (or flushing) away from the membrane surface [263,272]. Overall, even when operated at higher flux and enhanced fouling conditions, FO fouling is relatively easy to remove using osmotic backwashing [265]. However, enhanced fouling behaviours are expected when reaching high CF and especially with highly concentrated streams, but this impact has not been specifically evaluated in the literature, which typically operates fouling tests at low/moderate water recovery.

Concentration processes, especially those treating challenging streams, scaling, biofouling and clogging, should be carefully addressed. Scaling is the precipitation of sparingly soluble salts which occurs when reaching the limit of solubility. If mechanisms are similar to those of organic fouling, the occurrence and severity of scaling is strongly dependent on the concentration and solubility of potential scalants (or their precursors) in the feed solution and on the CF that is achieved [273]. Typical scaling in membrane processes is linked to calcium (calcium sulphate, calcium phosphate and calcium carbonate) or silica that may limit the recovery and concentration of the feed stream [24,274,275]. Scaling can also be enhanced by salt diffusion within the membranes (reverse or forward) that can locally increase the content of scaling ions and supersaturation [276,277]. Alkaline scale (calcium phosphate or carbonate formation) might be limited by the acidification of the solution [278]. Tuning the more appropriate draw solution might also mitigate scaling propensity.

Biofouling refers to the attachment and propagation of living microorganisms, present in the feed solution to the membrane surface, utilizing nutrients from the surrounding environment to form a biofilm which can colonize all the surfaces [24]. Biofilm formation can occur even with low initial amounts of microorganism, but is promoted when reaching high concentration, since nutrients are more concentrated. Biofouling can also be enhanced by the nutrients’ RSF from the draw solution [24]. In addition to the loss in permeation flux, biofouling can also lead to the biodegradation of nutrients from the feed solution, negatively impacting the effective concentration (of VFA, for example) [82]. Biofouling mitigation, especially when treating highly concentrated streams, is of key importance and should be mitigated by limiting filtration time, avoiding the presence of air and by implementing efficient biofouling mitigation cleaning [82].

Finally, the synergetic effects of organic fouling, scaling and biofouling were also observed, further enhancing cake formation and decreasing filtration [279,280,281]. All those effects were even more critical when concentrating feed solutions. This is a key aspect in the FO process concentration system, which requires a specific assessment. Additionally, most studies so far were performed at labs or in small pilot scale configurations for several hours up to several days of operation. Given the challenging stream to be concentrated, fouling phenomena will surely occur on a larger scale and longer-term operation. Clogging issues will certainly arise as well, but so far, they have not been considered in the literature. Assessing fouling and clogging issues is a major consideration to fully evaluate applications of FO in concentration processes, and requires specific work.

## 4. Concluding Remarks, Remaining Challenges and Future Research

Concentration appears to offer great opportunities for FO for a broad range of applications given its unique properties of high rejection rate and soft concentration process. Additionally, the advantage of FO relies on its possibility to concentrate complex streams of a broad range of compositions and initial concentrations and up to high concentration rates. Given those elements, FO appears to be more relevant and competitive where value is justified by the compounds to be concentrated, rather than by the water, which is extracted. This explains the current commercial push towards the recovery of high-value compounds (food and beverage, brine concentration, etc.) rather than water reuse and desalination processes. Still, several considerations remain towards successful future FO implementations.

### 4.1. Need for Dedicated Concentration Studies

This review also highlighted the fact that FO for concentration needs specific consideration at the fundamental level as well as requiring lab scale evaluation and full-scale validation. Most studies found in the literature were performed at bench scale using membrane coupons or small modules and with limited operation times (few hours to few days). Given the challenging stream to be concentrated, fouling phenomena will surely occur on a larger scale and during longer-term operation. Additionally, rather than evaluating the rejections of specific compounds, studies should consider the CF and defining limitations when reaching high concentration rates. Very rarely, studies were performed until the theoretical limit or requirements of the applications. Additionally, those studies need to consider not only the fraction of extracted water or the final concentration of the product but also analyse and report on the effective concentration of the compounds targeted as, due to imperfect rejection, poor recovery in biodegradation and deposition can be observed. Finally, operating until high CF will emphasize other potential limitations of FO, such as viscosity, enhanced fouling and clogging or scaling that will limit process viability and require proper technical answers, such as adapted cleaning procedure or required pre-treatment that has been rarely considered in the existing literature on FO concentration.

### 4.2. Design of FO Concentration Units

At the full scale level, FO offers a broad range of possibilities of operational design, such as feed and draw transfer in modules (co- or counter-current), module arrangements and module types (such as crossflow or submerged) and operation (in batches or one pass), which are key parameters that have to be further evaluated. One critical aspect of the FO concentration process is the design of units (feed and draw tanks, module arrangement) that will allow for appropriate operation during feed volume variations. This represents some challenges from lab scale to industrial design. CFD and numerical analyses will help to improve mass transfer, new module design and arrangements. Lab and small pilot scale experiments are also required to define the optimized operation parameters (levels of pre-treatment, optimized membranes, and module designs to favour high recovery and avoiding compounds losses or degradation).

Another important consideration is the draw solution. Operation with an available high osmotic pressure stream that does not require regeneration or recovery will largely favour FO economics; however, in most cases, this will not be possible and draw type and recovery system selection will need to be implemented. The testing and selection of draw solutions will have to better consider the level of final concentration desired, the suitability with applications (especially for food and beverages) and the associated solution for draw recovery.

### 4.3. To Demonstrate Competitiveness Versus Other Concentration Processes

As shown in Table 1, FO appears as a promising alternative to existing concentration processes versus competitive technologies. One key advantage is the operation at ambient temperature, which does not alter the organoleptic properties of the products to be treated, which is a key element, especially for temperature sensitive components (food and beverages, pharmaceuticals, etc.). Interestingly, also relying on osmotic pressure, high CF could be reached that is higher than RO and similar to the thermal processes. Energy needs in FO depends on draw recovery type, osmotic pressure required and the level of concentration to be achieved. There are some competitive advantages to be expected (especially in thermal processes), but they are still to be confirmed through pilot/full scale studies. If confirmed, FO becomes attractive for food/beverage applications as well as for ZLD and mining from waste streams. The potential in mining from wastewater combined with water reuse seems more challenging; however, full economic evaluation is still to be performed considering not only the benefits of water reuse but also savings in the WW treatment line. The availability/synergy with existing saline streams in that context, limiting draw recovery costs, is a key element to support FO implementation. Still, FO technology is only in its infancy, slowly reaching the market, and thus must demonstrate its potential in full scale, both in terms of technical performance and operation. 

## Figures and Tables

**Table 1:** Comparative properties and performances of concentration processes.

	UF/MF	NF/RO	MD/OD	Evaporation	Cryo-Concentration	Forward Osmosis
Temperature	Ambient	Ambient	Moderate	High	Low	Ambient
Pressure	Moderate	High	No	No	No	No
Concentration capability		Limited	High	High	Medium	High
Organoleptic preservation	Good	Good	Good–moderate	Poor	Good	Good
Applications	WW, food, waste streams, desalination	WW, food, waste streams. desalination	Desalination (MD)/food (OD)	Food, Desalination	Food	WW, food, waste streams, desalination
Energy	Moderate	High	Low–high	Very high	High	Low–high
Technology readiness level	Established technology	Established technology	Pilot scale	Established technology	Established technology	Reaching market

**Table 2:** Review of research studies of liquid food reporting concentration factor (CF) using cellulose triacetate (CTA) and thin-film composite (TFC) forward osmosis (FO) membranes.

Application	Membrane Type	Draw Solution	Initial Jw (L·m^−2^·h^−1^)	Initial Conc.	CF	Final Conc.	Ref.
Pineapple	CTA-FO	Sucrose/NaCl	<1	12°	5	60°	[44]
Kokum	CTA-FO	6 M NaCl	12.3	2°	26	52°	[45]
Tomato	TFC-RO	4 M NaCl	6	5.5°	2.9	16°	[53]
Grapefruit	TFC-FO	2 M NaCl	13	Pure juice	4	22°	[39]
Grapefruit	TFC-FO	2 M Glucose	13	Pure juice	4	22°	[39]
Grape	CTA FO	6 M NaCl	8.5	4.4°	12.3	54°	[46]
Beetroot	CTA FO	6 M NaCl	12.4	2.3°	22.6	52°	[46]
Pineapple	CTA FO	6 M NaCl	10.5	8°	6.8	54.6°	[46]
Raspberries	CTA FO		10.5	10°	4.5	45°	[41]
Sweetlime	CTA FO	6 M NaCl	9	11°	4.5	50°	[52]
Sucrose	CTA	4 M NaCl	5.8	10°	5.6	56.8°	[54]
Orange press liquor	CTA	4 M NaCl	16.8	8°	1.3	10.5°	[55]
Rose extract anthocyanin	CTA FO	6 M NaCl	11	4°	12.5	50°	[52]

**Table 6:** Types of commonly available draw solutes and their corresponding pros and cons (adapted from [257,258]).

Draw Solute Type	Examples	Draw Recovery	Advantages	Limitations
Inorganic salts	NaCl (or seawater), MgCl_2_, Na2SO4, (NH4)_2_SO_4_, KNO_3_	NF/RO, MD, etc. Direct use for fertilizers	High osmotic pressure Low cost (replenishment)	High RSD Divalent ions: scaling/fouling precursor NH_4_^+^, SO_4_^2−^, salinity: Biological process inhibitor
Organic salts	Zwitterions (amino acids) Organic ionic salts Hydroacid complex	NF, MD	Low RSD Recovery through NF Biodegradation in feed (OMBR, AnOMBR)	High cost (replenishment) Toxicity (?)
Organic coumponds	Sucrose, fructose, etc.	NF/RO	Suitable for food and beverages applications Recovery through NF	Low osmotic pressure
Stimuli responsive solutes (temperature, electricity, CO_2_, magnetic)	NH_4_HCO_3_	Distillation	High osmotic pressure	High RSD, Scaling precursor
	Ionic liquid, glycol ether, nanoparticles, polymer, switchable hydrogels	MF, magnetic field, phase separation, etc.,	Possibly lower energy for draw recovery	Cost, lab scale studies
